# Childhood Stress and Amygdala-Hippocampal Volume: A Multiverse Analysis in Adolescents

**DOI:** 10.64898/2026.10.13.26365419

**Published:** 2026-10-14

**Authors:** Parnika Sivakumar, Jamie Hanson

## Abstract

Childhood adversity is robustly linked to differences in amygdala and hippocampal volume. However, findings across studies have been inconsistent and contradictory. This inconsistency stems partly from the many reasonable but arbitrary choices that researchers make when operationalizing brain regions, stress exposure, and covariates. To deal with this variability, we applied a multiverse analysis approach using adolescent data from the Boston Adolescent Neuroimaging of Depression and Anxiety (BANDA) study to evaluate how multiple analytic choices influence the relationships between stress and hippocampal/amygdala volume. Our primary analysis crossed three region-of-interest operationalizations (left, right, total), four intracranial volume scaling methods, and multiple sociodemographic covariates. Greater lifetime stress severity was associated with smaller amygdala volume across 72% of specifications, and with smaller hippocampal volume across 52% of specification models. Statistical significance was strongly influenced by the region of interest. Our secondary multiverse analysis tested six operationalizations of stress exposure (total/acute/chronic x count/severity). This analysis found that the laterality pattern in the primary analysis was not strongly influenced by stress operationalization. These findings demonstrate the robustness of the stress-medial temporal lobe association and identify specific analytic choices (i.e. hemisphere operationalization) that most strongly drive the variability observed in previous literature. Our study is one of the first that utilized multiverse analysis for adversity-neuroimaging research, and provides a strong framework for evaluating and interpreting inconsistent findings.

